# Improved Drill Stock

**Published:** 1845-09

**Authors:** 


*Improved Drill Stock.*—Among the numerous improvements that have of late been made in dental instruments, is a drill stock, manufactured by Mr. Arnold of Baltimore. The improvement claimed by Mr. A. is, that while this instrument possesses all the advantages of the drill stocks now in use, it has the additional one of requiring but one hand of the operator to use it. It is certainly a very beautiful instrument, and there are many cases in which it may be advantageously employed.

